# Translation, Validation, and Cultural Adaptation of the Greek Versions of the Lysholm Knee Score and Tegner Activity Scale in Patients With Anterior Cruciate Ligament Injury

**DOI:** 10.7759/cureus.104823

**Published:** 2026-03-07

**Authors:** Apostolos D Prodromidis, Charalambos P Charalambous, Georgios C Thivaios, Dimitrios Erginousakis, Vasileios S Nikolaou, Jack Lysholm, Yelverton Tegner, Efstathios Chronopoulos

**Affiliations:** 1 Department of Trauma and Orthopaedics, 417 NIMTS (Army Equity Fund Hospital), Athens, GRC; 2 School of Medicine, National and Kapodistrian University of Athens, Athens, GRC; 3 Department of Trauma and Orthopaedics, Blackpool Teaching Hospitals NHS Foundation Trust, Blackpool, GBR; 4 School of Medicine and Dentistry, University of Lancashire, Preston, GBR; 5 Department of Trauma and Orthopaedics, Laiko General Hospital of Athens, Athens, GRC; 6 Department of Sports Injuries, KAT General Hospital, Athens, GRC; 7 2nd Academic Department of Orthopaedics, School of Medicine, Konstantopoulio General Hospital, National and Kapodistrian University of Athens, Athens, GRC; 8 Department of Health Sciences, Education and Technology, Luleå University of Technology, Luleå, SWE; 9 Department of Health Sciences, Medicine and Rehabilitation, Luleå University of Technology, Luleå, SWE; 10 Laboratory for Research of the Musculoskeletal System, KAT General Hospital, Athens, GRC

**Keywords:** acl injury, cultural adaptation, lysholm score, tas, tegner activity scale, translation

## Abstract

Background: The Lysholm score and the Tegner Activity Scale (TAS) are two knee-specific patient-reported outcome measures with widespread clinical and research use in patients after anterior cruciate ligament (ACL) injury. However, there is a lack of previously validated Greek-language patient-reported outcome measures for ACL-injured patients. The aim was to translate, cross-culturally adapt, and evaluate the psychometric properties of the Lysholm score and TAS in Greek for patients with ACL injury.

Methods: Translation and cross-cultural adaptation of Lysholm and TAS questionnaires into Greek, along with a full psychometric assessment of both questionnaires, were conducted using international guidelines. Convergent validity was assessed by determining their correlation to the Greek International Knee Documentation Committee - Subjective Knee Form (IKDC-SKF) and 36-Item Short Form Health Survey (SF-36) physical functioning subscale using Pearson’s correlation coefficient (r). Internal consistency of the Greek Lysholm score was determined using the Cronbach’s alpha coefficient (α). Test-retest reliability was examined in 40 patients drawn from the total study sample with the intraclass correlation coefficient (ICC) comparing the Greek Lysholm score and TAS values at baseline and around seven days later.

Results: The questionnaires were distributed to 98 adult patients with ACL injury (mean age: 25.5 years; 77 males). Forward-backward translations and cultural adaptation were smooth and consistent. Factor and Rasch analyses confirmed the unidimensional proposed factor structure of the Lysholm score. Both Lysholm and TAS showed strong correlation with the IKDC-SKF (r = 0.800 and r = 0.662, respectively) and the SF-36 physical functioning subscale (r = 0.713 and r = 0.597, respectively). There were no floor and ceiling effects. The Greek Lysholm score showed excellent internal consistency (α = 0.852), whilst both Lysholm and TAS had excellent test-retest reliability (ICC: 0.995 and 1, respectively).

Conclusions: The Greek-translated versions of the Lysholm score and TAS for patients with ACL injury are conceptually equivalent to the original versions with excellent cross-sectional validity and reliability. They are valid and reliable outcome measures for assessment of knee function and activity level in patients with ACL injury in Greece, and their use is recommended in clinical practice and research for ACL-injured patients in Greece.

## Introduction

Anterior cruciate ligament (ACL) tears represent a serious knee injury. They are particularly common in young adults, especially among football players, accounting for more than half of all sustained knee injuries [1]. The reported annual incidence of ACL injury is 36.9 per 100,000 in the general population and one per 1750 in the younger population [1]. The ACL is one of the main anteroposterior and rotational stabilizers of the knee [2]. Most ACL injuries occur mainly through non-contact mechanisms, such as abrupt changes of direction, sudden stops, rapid acceleration, or incorrect landing after a jump, and less commonly due to direct contact injuries, such as a lateral impact to the knee [3]. Patient-reported outcome measures (PROMs) aim to assess the patients’ perspective in relation to various aspects of health, such as knee symptoms and function, as well as assess the patient’s quality of life [4]. Knee PROMs and rating scales have been developed to assess outcomes following ACL injury or ACL reconstruction surgery, and also to assess the injury severity and recovery [5].

The Lysholm score and the Tegner Activity Scale (TAS) are two well-established PROMs with widespread clinical and research use to assess outcomes in patients with ACL injury [6,7]. The Lysholm score was designed to evaluate the functional status of the knee in patients with ACL injury. It is an eight-item questionnaire asking patients to evaluate how much each domain is affected, with a total score up to 100: (i) limp; (ii) using cane or crutches; (iii) locking; (iv) giving way/instability; (v) pain; (vi) swelling; (vii) climbing stairs; (viii) squatting. The TAS, published in 1985 as an extension of the Lysholm score, was developed to evaluate activity level only based on occupational and sports activities. It is a one-item questionnaire asking patients to evaluate their current activity level for competitive or recreational sports and occupational activities on an 11-point scale.

Both the Lysholm score and TAS have been extensively used, and subsequent studies have established their validity and reliability for patients with ACL injury [8]. They both have been validated and culturally adapted for use in patients with ACL injury into several languages across the world [9-11]. It is crucial to test the psychometric properties of a self-reported PROM rather than only translate its content, for it to be a valid and reliable tool in populations with different cultures [12]. Validity is the most critical psychometric domain when adapting PROMs across cultures, as it shows how well an instrument/questionnaire measures what it is supposed to measure, and it measures accuracy, while reliability measures consistency. The aim of cross-cultural adaptation is to produce a target questionnaire/tool equivalent to the source questionnaire based on its content [13].

Despite extensive, high-quality research outputs in Greece focusing on ACL injuries, there is a lack of previously validated Greek-language PROMs for ACL-injured patients. A translated version of the Lysholm score and TAS published in 2020 with a basic psychometric assessment was for patients with a variety of knee pathologies and did not focus on ACL injuries, as per the original design of these questionnaires [14]. A validated and cross-culturally adapted Greek version of both Lysholm and TAS questionnaires for patients with ACL injury would provide Greek clinicians and researchers with a valid and reliable PROM to evaluate the progress of these patients and to safely compare results.

This study aimed to translate and cross-culturally adapt the original English versions of the Lysholm score and TAS into the Greek language and perform a psychometric evaluation focused on cross-sectional validity and reliability of the translated versions in patients with ACL injury.

## Materials and methods

Study design, setting, and participants

A methodological instrument validation study with a cross-sectional design and a short-term longitudinal component (test-retest reliability) was conducted from April 2022 to June 2024 at the KAT General Hospital of Athens in Greece. Ethical approval from the KAT Hospital’s Ethical Committee (ethical approval protocol: 2767/03-03-2022) was obtained, and all participants signed a written informed consent for participation in the study.

The study was performed according to published international guidelines for translation and cross-cultural adaptation and measurement properties of health questionnaires, as described by Beaton et al. and COSMIN (Consensus-Based Standards for the Selection of Health Measurement Instruments), respectively [13,15]. Due to a lack of qualitative criteria for the assessment of the sample size, we used the most commonly reported ratios in the bibliography. The sample size required for the confirmatory factor analysis (CFA) was based on researchers' conventions ranging from 4:1 to 12:1 (participants to variables) ratio [16]. For the exploratory factor analysis (EFA), a sample size adhering to the commonly recommended ratio of 10 participants per variable was used [17]. The Lysholm questionnaire consisted of eight items; thus, our sample size of 98 participants is within the above guidelines. The Kaiser-Meyer-Olkin measure of sampling adequacy was also used to confirm the suitability of the data for factor analysis [18].

Eligibility criteria for patients were as follows: (i) skeletally mature patients (age > 16 years old) aged 16 to 18 years old who gave consent with one parent present and both signed the consent; (ii) patients in Greece whose primary language is Greek; (iii) diagnosis of ACL injury as determined by magnetic resonance imaging (MRI); (iv) patients with an isolated complete ACL injury without other concomitant injuries; (v) patients awaiting ACL reconstruction completing the questionnaires strictly pre-operatively.

Patients were excluded if they had (i) concomitant injuries such as meniscal tear, collateral ligament tear, or patellar dislocation, and (ii) a history of recent lower limb surgery (other than ACL reconstruction).

Translation and cross-cultural adaptation

**Figure 1:**
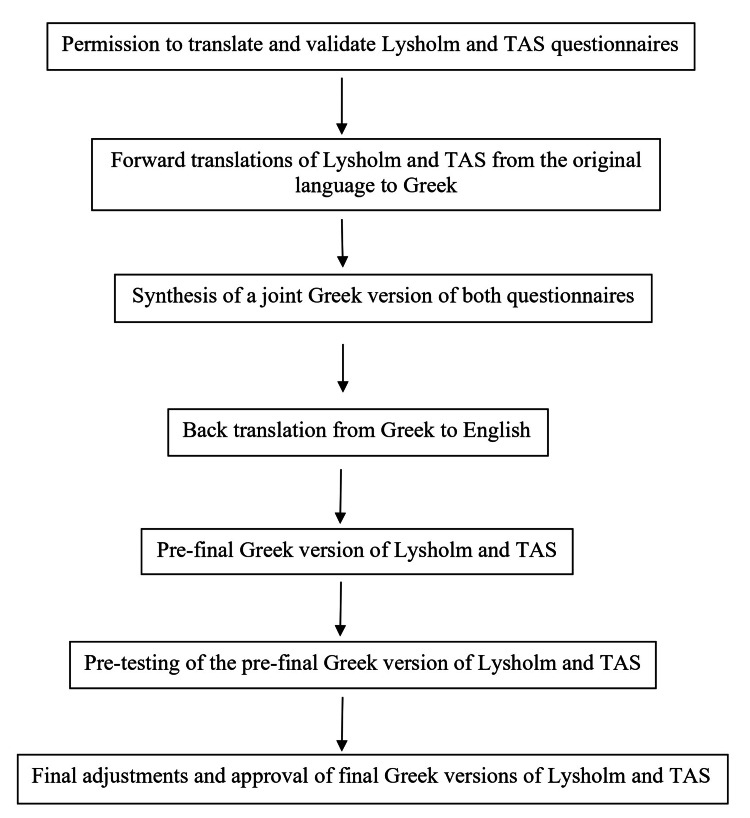
Translation and adaptation steps of our protocol according to international guidelines. Guidelines: Beaton et al. [13]. TAS: Tegner Activity Scale.

The validation and cross-cultural adaptation of the original English version of both the Lysholm score and TAS into the Greek language were performed according to international recommendations and guidelines as set by Beaton et al. and COSMIN [13,15]. It included the following steps: (1) the original creators of the questionnaires were contacted, and permission to translate and validate both the Lysholm and TAS questionnaires was obtained. (2) Forward translation of both the Lysholm score and TAS from the original (English) language to Greek by two independent bilingual (Greek native) translators with medical backgrounds was then performed (ADP, EC). (3) Consensus meeting: Discussion between the two translators of any discrepancies and synthesis of a joint version of the translation in Greek for both questionnaires. (4) Back translation by a bilingual (English native) translator with a medical background (CPC). (5) Consensus meeting: All translators evaluated the back-translations and checked for inconsistencies with the original English versions for any ambiguities and other expression issues, before producing the pre-final Greek versions for both Lysholm and TAS. (6) Pre-testing: Pilot distribution of the pre-final Greek versions for both Lysholm and TAS. A total of 33 patients were recruited for the pilot study in line with previous guidelines [13]. They completed the pre-final Greek versions in the presence of a researcher for assessment, and feedback was obtained regarding the clarity of the questions and answer options with an informal discussion. No item required modification following feedback. (7) Final adjustments according to patient feedback obtained in the pilot distribution and approval of the final Greek versions of Lysholm and TAS (Appendices). The translation and adaptation steps of our protocol are summarized in Figure 1.

Questionnaires

For this study, patients completed the following questionnaires: (i) Greek version of the Lysholm score (Appendix A); (ii) Greek version of the TAS (Appendix B); (iii) Greek validated version of the International Knee Documentation Committee (IKDC)-Subjective Knee Evaluation Form (SKF) [5,19]; (iv) Greek validated version of the 36-Item Short Form Health Survey (SF-36) [20,21].

Psychometric assessments and statistical analysis

Construct validity was determined using the convergent validity [22] and the item response theory (IRT) Rasch model analysis [23,24]. Convergent validity of the Greek version scores was based on a theoretically grounded hypothesis testing predicting strong correlations with the IKDC-SKF and the SF-36 physical functioning subscale. Pearson’s correlation coefficient (r) was used to determine the correlation that would support the validity of the Greek version of the questionnaires to measure functional ability and activity level, respectively. The Greek versions of IKDC-SKF and the physical functioning subscale of the SF-36 were used specifically as they focus on the level of function and activity, same as the Lysholm and TAS questionnaires. Rasch model analysis was performed for the Greek version Lysholm score. The TAS scale is a single-item ordinal measure, which precludes the assessment of key Rasch model properties such as internal consistency, person and item separation reliability, and verification of unidimensionality through residual analyses. Hence, Rasch analysis was not performed for the assessment of the validity of the TAS.

Item polarity, item fit, separation index, person reliability, and item reliability were used to evaluate the validity of the Lysholm score [24,25]. Acceptable fit criteria [23,24]: (i) for item polarity, the point measure correlation (PTMEA CORR) must be > 0; (ii) for item fit, the mean square (MNSQ) infit and outfit must be within 0.6 to 1.4, respectively; (iii) for separation value, all items must show value ≥ 2.0; (iv) for item reliability and person reliability, both must be > 0.8. The Rasch measurement model also provides evidence about unidimensionality through principal component analysis (PCA) on the residual score [26]. The two PCA indicators of unidimensionality are the raw variance explained by measures (RVEM) and unexplained variance in first contrast (UVFC) [26]. Indicators for unidimensionality [23,24,26]: RVEM must be > 40.0% and UVFC must be < 5% with an eigenvalue of < 3.0.

EFA was performed to assess the factor structure of the eight items of the Lysholm score, applying the maximum likelihood extraction method with Varimax rotation [27]. CFA was conducted using the Analysis of Moment Structure (AMOS) version 21.0 to assess the factor structure of the Lysholm questionnaire as suggested by its author [28]. Accepting a model was based on specific global fit indices: (i) chi-square degrees of freedom (d.f.) ratio < 2.0; (ii) root mean square error of approximation (RMSEA) < 0.08; (iii) comparative fit index (CFI) > 0.90; (iv) normed fit index (NFI) > 0.90; (v) goodness fit index (GFI) > 0.85; (vi) adjusted GFI (AGFI) > 0.80.

Known groups validity was also assessed to explore whether the Greek version of the Lysholm score could distinguish different patient groups based on their functional ability according to TAS [22]. Looking at the TAS 11-level scale and with the lack of evidence regarding TAS cut-off values, we predefined the TAS level of 4 as a reasonable cut-off to differentiate between low or moderate and high levels of activity.

Item analysis was performed for the Greek version of the Lysholm score through the item discriminating power and the item difficulty.

Floor and ceiling effects (FCEs) and minimal important change (MIC) were used to assess interpretability. If more than 15% of participants scored the lowest or highest possible scores, FCEs were considered to exist [29]. MIC is the smallest difference in score that patients perceive as clinically meaningful, and it was defined as 0.5 x standard deviation (SD) at baseline [29].

Measurement error was assessed by measuring the standard error of measurement (SEM) and minimal detectable change (MDC), which is the smallest change in the score that can be interpreted as a real change beyond SEM.

Internal consistency reliability of the Greek version of the Lysholm score was assessed with the Cronbach’s alpha coefficient (α), with a value > 0.7 indicating adequate reliability in research [30]. Test-retest reliability was examined in a sample of 40 patients drawn from the total study population cohort, comparing the Greek Lysholm score and TAS values at baseline and around seven days later (median IQR: 7; range: 6-9; 95% CI: 5.9-8.7) through the intraclass correlation coefficient (ICC) [31]. As ICC does not correct for systematic differences and random agreement, the scores of the two assessments were tested for systematic differences using the paired t-test. Finally, the Bland-Altman plot was used to assess stability visually [32,33].

Differential by sex or surgical status was not assessed as the sample size was relatively small for such an analysis.

Analyses were carried out using SPSS version 21.00 (IBM Corp., Armonk, NY), the Winsteps Rasch Measurement Software version 3.81.0 (Winsteps, Beaverton, OR), and the MedCalc® Statistical Software version 20 (MedCalc Software Ltd, Ostend, Belgium).

## Results

Participants

Data were collected for 98 patients, a sample that was within the relevant guidelines used [13], and within the most commonly reported ratios in the bibliography for factor analysis [16,17]. Patients’ mean age was 25.5 years (SD: 6.8), with 77 males and 21 females. The mean Lysholm score of the participants was 75 (SD: 22.8), and the median (min-max) TAS score was 3 (0-9). All the demographic and clinical characteristics of participants are summarized in Table 1.

**Table 1:** Demographic and clinical characteristics of the participants. ACL: anterior cruciate ligament; SD: standard deviation; min: minimum; max: maximum; BMI: body mass index; Hx: history.

Characteristic	Value
Gender: Male/Female, n (%)	77 (78.6)/21 (21.4)
Age (years): Mean ± SD (min-max)	25.5 ± 6.8 (16-48)
Weight (kg): Mean ± SD (min-max)	82.7 ± 11.7 (57-110)
Height (cm): Mean ± SD (min-max)	180.0 ± 6.3 (168-196)
BMI: Mean ± SD (min-max)	25.5 ± 3.2 (18-38)
Family status: Married/Single, n (%)	18 (18.4)/80 (81.6)
Education: High school/University, n (%)	51 (52.0)/47 (48.0)
Injured leg: Right/Left, n (%)	55 (56.1)/43 (43.9)
Smoking: No/Yes, n (%)	80 (81.6)/18 (18.4)
Surgery: No/Yes, n (%)	78 (79.6)/20 (20.4)
Hx of contralateral ACL injury: No/Yes, n (%)	92 (93.9)/6 (6.1)
Greek Lysholm score: Mean ± SD (min-max)	75.0 ± 22.8 (0-100)
Greek Tegner score: Median (min-max)	3.0 (0-9)

Translation and cross-cultural adaptation process

Translations were smooth, with backward translations being consistent with the original English versions, and no major modifications were required. However, minor adjustments were necessary for the translated TAS regarding sports not popular in Greece (rugby, ice hockey, racquetball, and squash), with the removal of these. In line with COSMIN guidelines [15], we removed rugby and ice hockey from higher activity levels (9 and 10). For intermediate activity levels 6 and 7, we removed racquetball but kept tennis, which is the equivalent popular sport in Greece. For levels 7 and 8, we also removed squash but kept badminton, as Greek people are more familiar with the latter.

Feedback and assessments during the pilot study showed conceptual equivalence between the Greek and the original questionnaires. There were no issues with clarity and acceptance of items.

Psychometric assessments

Construct validity was assessed as previously referenced. Table 2 summarizes the results. Convergent validity was satisfied as there was a very strong correlation between Lysholm total score and IKDC-SKF score (r = 0.800, p < 0.005) and SF-36 physical functioning subscale (r = 0.713, p < 0.005), and a strong correlation between TAS and IKDC-SKF score (r = 0.662, p < 0.005) and SF-36 physical functioning subscale (r = 0.597, p < 0.005).

**Table 2:** Convergent validity of the Greek versions of the Lysholm score and TAS. Correlations were presented as Pearson’s correlation coefficient (r). Cut-off points for r: 0.10-0.30 (weak); 0.30-0.50 (moderate); 0.50-0.70 (strong); 0.70-0.90 (very strong) [34]. TAS: Tegner Activity Scale; IKDC: International Knee Documentation; SKF: Subjective Knee Form; SF-36: 36-Item Short Form Health Survey.

	Lysholm total score (r)	p-value	TAS (r)	p-value
IKDC-SKF total score	0.800	<0.0005	0.662	<0.0005
SF-36, Physical functioning	0.713	<0.0005	0.597	<0.0005
SF-36, Role physical	0.169	0.095	0.277	0.006
SF-36, Bodily pain	0.532	<0.0005	0.501	<0.0005
SF-36, General health	0.192	0.058	0.086	0.397
SF-36, Vitality	0.515	<0.0005	0.422	<0.0005
SF-36, Social functioning	0.583	<0.0005	0.445	<0.0005
SF-36, Role emotional	0.312	0.002	0.300	0.003
SF-36, Mental health	0.218	0.032	0.337	0.001

Data from the Greek Lysholm questionnaire were fitted to the Rasch partial credit model to perform an IRT Rasch model analysis (Table 3). Items were ordered according to their level of difficulty, and item 8 (squatting) was the most difficult, whereas item 5 (pain) was the easiest to answer. The reliability of test items and respondents for the Greek Lysholm score, and also their separation, was high and within limits. The PTMEA CORR values, which measure the item polarity for all eight items, ranged from 0.60 to 0.76. The reported unidimensionality of the Greek Lysholm questionnaire was good (RVEM = 55.2%, > 40%; UVFC = 2.9%, < 5%, with an eigenvalue of UVFC = 1.8, < 3).

**Table 3:** Fit of admission Lysholm score items to the Rasch model. Rasch model analysis was performed using the Winsteps Rasch Measurement Software version 3.81.0. Acceptable fit criteria [23,24]: For item fit: infit-outfit MNSQ must be 0.6-1.4, respectively; for item polarity: PTMEA CORR must be > 0; for separation value: items must show ≥ 2.0; item and person reliability must be > 0.8. Indicators for unidimensionality [23,24,26]: RVEM > 40.0% and UVFC < 5%; UVFC < 5% and eigenvalue < 3.0. SE: standard error; MNSQ: mean square; PTMEA CORR: point measure correlation; RVEM: raw variance explained by measures; UVFC: unexplained variance in first contrast.

Lysholm score item	Measure	SE	Infit MNSQ	Outfit MNSQ	PTMEA CORR
Lysholm 8: Squatting	61.16	0.67	0.61	0.67	0.61
Lysholm 1: Limp	58.55	0.69	0.61	0.95	0.61
Lysholm 2: Using cane or crutches	56.14	0.65	0.58	0.56	0.60
Lysholm 6: Swelling	51.54	0.49	0.95	1.14	0.69
Lysholm 7: Climbing stairs	51.16	0.48	0.84	1.13	0.73
Lysholm 3: Locking sensation	45.60	0.39	0.93	1.20	0.74
Lysholm 4: Giving way sensation	38.80	0.36	1.23	1.33	0.76
Lysholm 5: Pain	37.05	0.38	1.31	1.39	0.76
Criterion	Reliability			Separation	
Person	0.90			2.93	
Item	0.99			15.64	
Unidimensionality					
Measures				Value	
Raw variance explained by measure (RVEM)				55.2%	
Unexplained variance in first contrast (UVFC)				2.9%	
Eigenvalue of UVFC				1.8	

EFA was conducted as previously referenced. The Kaiser-Meyer-Olkin measure of sampling adequacy (0.894) showed a high level of sampling adequacy for factor analysis [18]. Furthermore, Bartlett’s test of sphericity was statistically significant (χ2 = 424.2, d.f. = 28, p <0.001), rejecting the hypothesis of an identity correlation matrix and indicating sufficient correlations among the variables. One factor with an eigenvalue of over 1 and item factor loadings greater than or equal to 0.40 was identified (Table 4). The eigenvalue for the first factor was 4.8, explaining 60% of the variance. Factor loadings, which are the correlation coefficients between the items and the factor, ranged from 0.65 to 0.85.

**Table 4:** Test-retest reliability in 40 patients (a) comparing scores at baseline and approximately seven days later using the paired-samples t-test for the Lysholm total score and the Wilcoxon signed-rank test for the TAS score, and (b) using the intraclass correlation coefficient (ICC) to assess the correlation between the two measurements. ^a^: Paired samples t-test; ^b^: Wilcoxon test; TAS: Tegner Activity Scale; ICC: intraclass correlation coefficient; 95% CI: 95% confidence interval; SD: standard deviation.

Scores	ICC (95%CI)	p-value	Baseline	Reassessment	t-value/z-value	p-value
			Mean ± SD			
Lysholm total score	0.995 (0.99-1.00)	<0.0005	73.75 ± 17.03	73.90 ± 16.56	t-value (_39_): 0.583	0.563^a^
TAS	1 (1.00-1.00)	<0.0005	3.10 ± 1.80	3.10 ± 1.80	z-value: 0.000	1.000^b^

**Figure 2:**
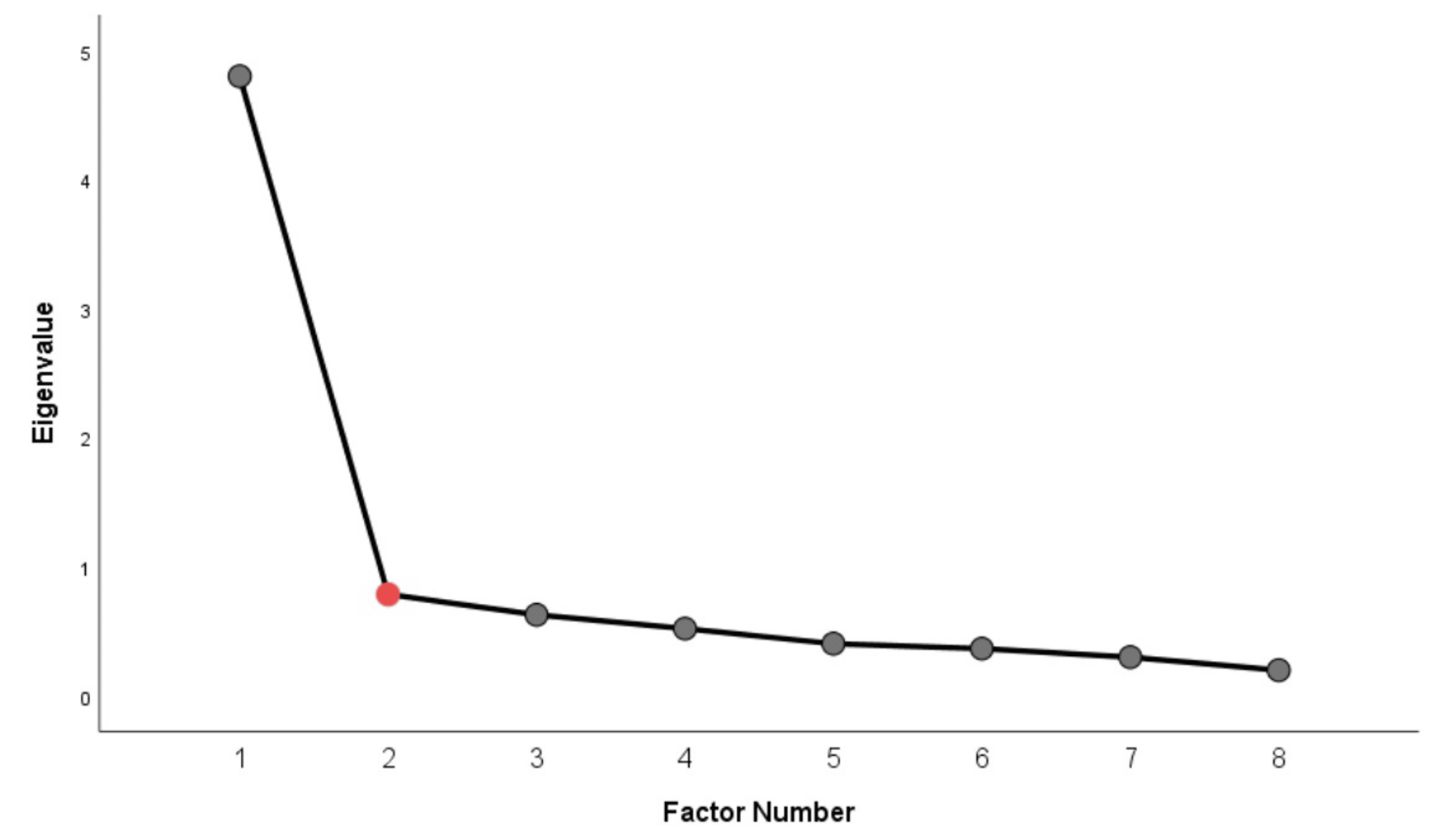
Scree plot of exploratory factor analysis. The scree plot displays eigenvalues plotted against factor number and is used to determine the number of factors to retain. Factors preceding the point of inflection (“elbow”) were retained, as they account for substantial shared variance; subsequent factors contribute minimal additional information.

The scree test (Figure 2) and Monte Carlo PCA for parallel analysis indicated one factor structure (the second eigenvalue of 1.27 was higher than the eigenvalue of the second factor, which was 0.79).

A unifactorial model of the original Lysholm score was assessed by CFA, reporting acceptable global fit indices. The resulting global fit indices (X2 = 47.4, d.f. = 24, chi-square degrees of freedom (d.f.) ratio = 1.98, RMSEA = 0.082 (90% CI: 0.050-0.114), CFI = 0.934, NFI = 0.893, GFI = 0.886, AGFI = 0.805) were all acceptable, with five out of the seven indices being within acceptable limits. RMSEA and NFI indices were borderline but very close to the acceptable thresholds, with RMSEA of 0.002 above the threshold and NFI of 0.007 under the threshold. These indices showed that the unidimensionality suggested by its creator should be accepted.

Regarding known-groups validity, the Greek Lysholm total score well distinguished different groups of patients based on their functional ability according to TAS. Lysholm total score was statistically significantly higher for patients with TAS score ≥ 4, as compared to those with TAS score < 4 (78.77 ± 14.15 vs. 64.36 ± 24.20, respectively, p < 0.005 - independent samples t-test).

The results of the item analysis for the Greek Lysholm score showed difficulty indices of the eight items ranging between 0.58 and 0.82. The easiest item to perform was “Item 2: Using cane or crutches” (0.82), while the most difficult item was “Item 8: Squatting” (0.58). Coefficients > 0.28 are considered to show satisfactory discrimination property. The range of Lysholm item discriminative indices was 0.58 to 0.76. “Item 1: Limp” was the most discriminative item (r = 0.76), while “Item 8: Squatting” was the least discriminative item (r = 0.58). A total of 4.1% of patients scored the lowest possible Lysholm total score, while 2% of patients scored the highest score. The respective lowest possible score percentage for TAS was 5.1%, and the highest was 2%. Since these percentages were far lower than the critical value of 15%, there was neither a floor nor a ceiling effect. The MIC value was 11.4, which means that an improvement of ≥ 11.4 points shows clinically significant improvement, whereas a smaller change may not be noticeable or clinically relevant.

The SEM was also determined, and the error associated with the Lysholm total score was 1.02, and the corresponding MDC value was 2.83.

The internal consistency reliability of the Greek Lysholm total score was estimated as 0.852, which indicates excellent internal consistency for the total score. For the test-retest reliability, the paired samples t-test between the initial assessment and reassessment of the Greek Lysholm total score and TAS showed no statistically significant difference. ICC between initial assessment and reassessment of the Lysholm total score and TAS was 0.995 (p < 0.005) and 1 (p < 0.005), respectively (Table 4).

**Figure 3:**
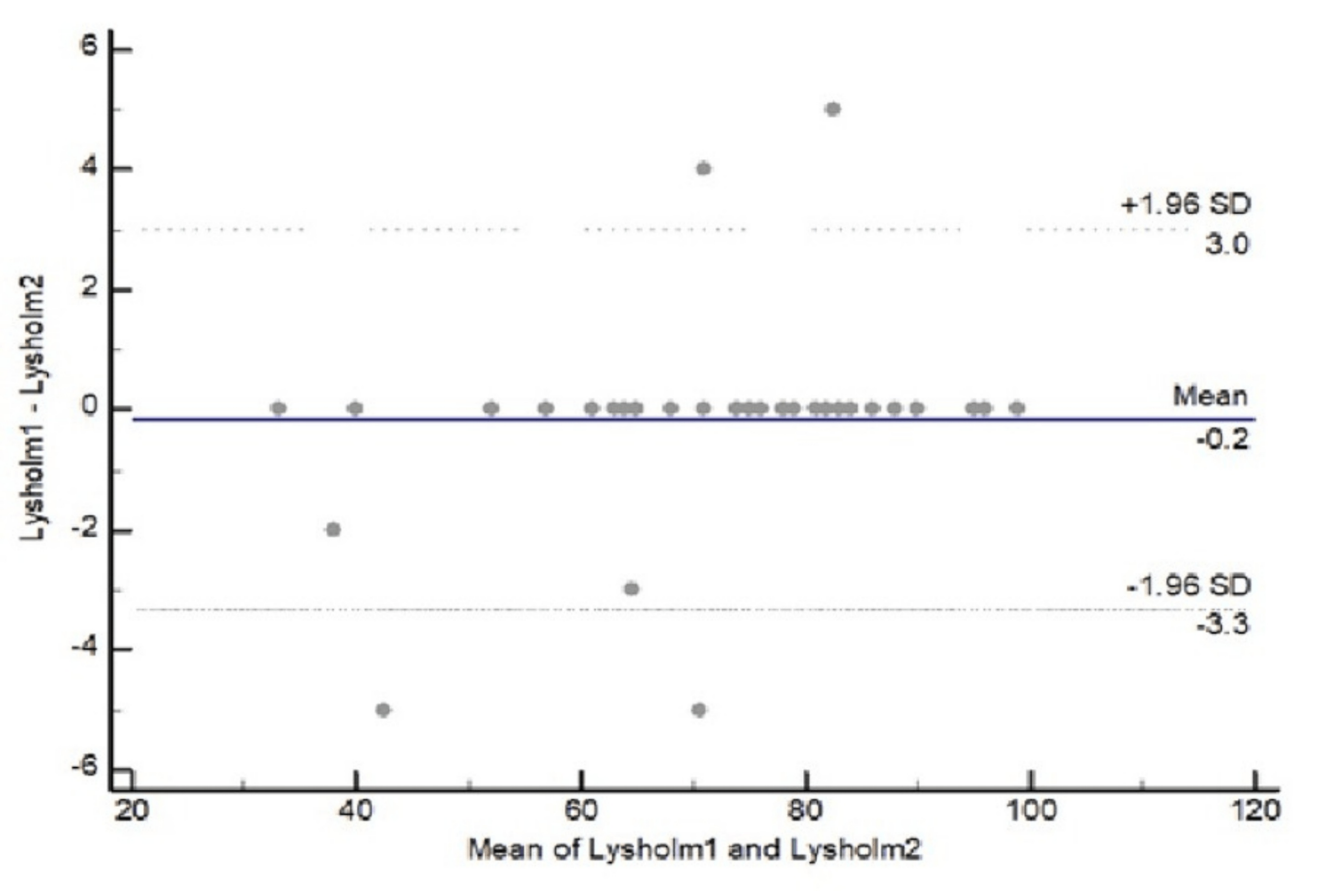
Bland-Altman plot of Lysholm total score assessing agreement between two measurements of the same population (mean difference: -0.2; 95% CI: -3.3 to 3.0). The solid line represents the mean difference (bias), and the dashed lines indicate the limits of agreement (±1.96 SD). A small mean difference (bias) and narrow limits of agreement indicate good agreement, whereas a large mean difference (bias), wide limits, or the presence of trends indicate poor or value-dependent agreement [32].

Bland-Altman plot of Lysholm total score, which is presented in Figure 3, showed agreement between assessments with differences being within mean difference ± 2 SDs; hence, the Greek Lysholm total score was consistent at baseline and around seven days later.

## Discussion

This study presents the cross-cultural adaptation and validation of the Greek versions of the Lysholm score and TAS for patients with ACL injury or post ACL surgery. The translation and psychometric properties testing were performed following established international guidelines for health-related instrument validation [13,15]. The results of this study showed that the Greek versions of the Lysholm score and TAS have very good psychometric performance in terms of cross-sectional validity and reliability to be used for Greek patients with ACL injury.

The Lysholm and TAS were originally developed in the English language with English-speaking patients and have since then been translated to multiple languages with good results [9-11]. Our findings are in line with those studies, as these scores translated and culturally adapted to a Greek population with ACL injury are equivalent to the original English version and have very good psychometric properties. However, simply translating a health-related questionnaire is not adequate, as its previous validation may not apply to a different population, culture, and context. Therefore, standardized international protocols for translation of such questionnaires exist to ensure valid translation quality and contribute to better responsiveness and reduced sampling error [13,15]. Following translation, psychometric evaluation of the questionnaire needs to be performed in the target population, a process known as cross-cultural adaptation [15]. Cross-cultural adaptation aims to provide equivalence between the translated and the original questionnaire based on content and reduce the risk of introducing bias. The standardized international protocols that were used in our study incorporate the principles of cross-cultural adaptation [13,15]. In line with those protocols, we removed sports that are uncommon in Greece (such as rugby, ice hockey, racquetball, and squash) and kept others of similar intensity for that level of activity, which are familiar to Greek people. Hence, the Greek TAS kept its meaning and its anchors for each activity level.

Evaluating the convergent validity of the Greek versions of the Lysholm score and TAS, it yielded statistically significant, highly positive correlations with the IKDC-SKF and the SF-36 physical functioning subscale [19,20]. Convergent validity is situated within the construct validity and is based on theoretically grounded hypotheses predicting strong associations with related measures, without requiring an absolute (“gold”) standard [22]. Convergent correlations, while strong, do not constitute a true gold standard comparison, but they support the validity of both the Greek versions of the Lysholm and TAS as measures of knee-related functional status and activity level. Rasch model analysis reported good unidimensionality of the Greek Lysholm score; hence, the unidimensional proposed factor structure of the Lysholm score was confirmed for the Greek sample. The TAS is a single-item ordinal measure, which precludes the assessment of key Rasch model properties such as internal consistency, person and item separation reliability, and verification of unidimensionality through residual analyses. Furthermore, previous findings have indicated that the response categories of the TAS may exhibit disordered thresholds, suggesting that respondents do not consistently distinguish between adjacent activity levels, thereby violating core assumptions of the Rasch model. Finally, potential differences in the interpretation of activity levels across populations cannot be adequately examined due to the single-item structure of the scale, limiting the appropriateness of Rasch analysis as a primary psychometric validation method for the TAS. Due to these important methodological limitations, Rasch analysis was not performed for the assessment of the construct validity of the TAS.

The Greek Lysholm total score well discriminated between different groups of patients based on their functional status according to TAS. There is a lack of evidence and no consensus regarding the definition of low and high activity levels according to TAS. Hence, looking at the TAS 11-level scale, we selected the TAS level of 4 as a reasonable cut-off point to differentiate low or moderate from high levels of activity.

Furthermore, there were no FCEs for both Greek version scores, with percentages much lower than the 15% needed to represent FCE. Similarly, in other validation studies for use of the Lysholm score and TAS in the Netherlands and China [10,11], the relevant translated scores showed acceptable FCEs. These findings across different cultures indicate good overall homogeneity of the Lysholm and TAS scores in different cultures and populations.

The internal consistency reliability of the Greek Lysholm total score was high (α: 0.852). Similar results of good internal consistency have been published for translated versions of the Lysholm score into the Spanish and Dutch languages [9,10]. The Greek Lysholm score and TAS also showed excellent test-retest reliability between repeated assessments within seven days (ICC: 0.995 and 1, respectively). Test-retest reliability for both the Lysholm score and TAS has been consistently reported as excellent in the literature in other studies of translated Chinese [11], Dutch [10], and Spanish versions [9].

Regarding the time interval for the assessment of test-retest reliability, there is no consensus on the most appropriate interval, and in our study, an interval of around seven days was considered the most relevant. Generally, most researchers select a time interval of two days to two weeks for assessment of test-retest reliability, with two weeks being the most popular [35]. However, such a long interval may not be achievable and yields a high risk of attrition bias. Protocols that use seven to 10 observation days for the participants obtain more reliable measurements of key physical activity metrics [31]. On the other hand, a short time interval increases the likelihood of carryover effects but reduces the risk of recall bias and drop-outs [35]. Hence, we selected a short time interval during which the participants remained clinically stable and untreated to balance between recollection bias and unwanted change in the patient’s clinical condition, as a longer interval could allow a change in symptoms with further injury occurring. The short time interval also explains the excellent ICC, since it is reasonable for the participants to give similar answers and report almost no change in their answers, resulting in an ICC of 1.

This study has its own limitations. The main limitation is that the responsiveness of the Greek version scores was not assessed in this study. Therefore, future research could focus on the responsiveness of these scoring systems and their sensitivity to detect relevant clinical changes over time, especially during rehabilitation or post-reconstruction surgery. Another limitation is that this study included only patients with isolated ACL injury and excluded patients with other associated intra-articular knee injuries, such as meniscal tears or collateral ligament injuries. The inclusion of patients with isolated ACL injury increases the homogeneity of the sample and the internal consistency but limits the possibility of generalizing the results and the application of these scoring systems to other knee joint pathologies. Hence, the Greek Lysholm and TAS may require additional validation in populations with associated knee injuries. Also, sampling was not stratified by gender, but included consecutive patients seen in the clinic. Although our sample showed gender asymmetry with predominance of male patients, the Lysholm questionnaire is not gender-specific. Therefore, the psychometric validation presented in our study remains valid, but generalizability to the underrepresented gender and age (female or older patients) may be limited. However, ACL injury is generally predominant in younger athletic patients [1]; hence, age is not a limitation. However, as ACL injury is common in female athletes, sex may have to be the focus of future research to assess if it affects the psychometric performance of these questionnaires.

Furthermore, a study by Panagopoulos et al. in 2020 reported the Greek versions of the Lysholm and TAS [14], but their wording differs substantially from our Lysholm and TAS versions in many of the questions and answer options. We feel that the Greek versions of our questionnaires are ACL-specific and more valid adapted tools to be used, such as for ACL-injured patients in Greece. Although Panagopoulos et al. followed similar principles to ours for the translation and cross-cultural adaptation, they performed a basic psychometric assessment. Our psychometric evaluation is deep and thorough, with the basic assessments of internal consistency, convergent validity, and test-retest reliability, but also with further analysis performing Rasch model analysis, exploratory and confirmatory factor analysis, item analysis, and measuring MIC, MDC, and SEM. Pre-testing of our questionnaires was performed in a much bigger sample (33 patients) following guidelines for the cross-cultural adaptation of PROMs, which recommend performing the pre-test in a sample of 30-40 patients [13]. For the Greek version of the Lysholm score, we adhered to the expressions used in the original English questionnaire to deliver the same meaning and kept the pain scale lines. Furthermore, Lysholm and TAS were originally designed for isolated ACL injuries and were not intended to evaluate other knee pathology. Hence, we included only patients with isolated ACL injury and no other associated knee pathology.

## Conclusions

In conclusion, the Greek-translated and culturally adapted versions of the Lysholm score and TAS scale for patients with ACL injury are conceptually equivalent to the original, with excellent cross-sectional validity and reliability. Therefore, the Greek versions of the Lysholm score and TAS are valid and reliable outcome measures for assessment of knee function and activity level, respectively, in patients with ACL injury in Greece whose primary language is Greek. Their use is recommended in clinical practice and research for ACL-injured patients in Greece.

## Appendices

Appendix A: Greek version of the Lysholm score

**Table 5:** Greek Lysholm score. Παρακαλώ βάλτε ένα σημάδι πάνω στη γραμμή για να δείξετε το μέγεθος του πόνου που είχατε στα γόνατα σας τις τελευταίες 24 ώρες: ΔΕΞΙΟ ΓΟΝΑΤΟ Καθόλου πόνος ------------------------------------------------------------------------ Χειρότερος δυνατός πόνος ΑΡΙΣΤΕΡΟ ΓΟΝΑΤΟ Καθόλου πόνος ------------------------------------------------------------------------ Χειρότερος δυνατός πόνος

ΕΡΩΤΗΜΑ\ΤΟΛΟΓΙΟ LYSHOLM Αυτό το ερωτηματολόγιο έχει σχεδιαστεί για να δώσει στο θεραπευτή σας πληροφορίες σχετικά με το πώς τα προβλήματα με το γόνατο σας έχουν επηρεάσει την ικανότητα σας να ανταπεξέρχεστε στην καθημερινή ζωή. Παρακαλώ απαντήστε κάθε ενότητα και σημειώστε μόνο εκείνο το ΕΝΑ κουτάκι που ταιριάζει καλύτερα σε εσάς αυτή τη στιγμή.	
ΕΝΟΤΗΤΑ 1: ΔΥΣΧΕΡΕΙΑ ΣΤΗ ΒΑΔΙΣΗ (ΧΩΛΟΤΗΤΑ)	Δεν κουτσαίνω όταν περπατώ. (5) Κουτσαίνω ελαφρώς ή περιοδικά όταν περπατώ. (3) Κουτσαίνω πολύ και συνεχώς όταν περπατώ. (0)
ΕΝΟΤΗΤΑ 2: ΧΡΗΣΗ ΜΠΑΣΤΟΥΝΙΟΥ ή ΠΑΤΕΡΙΤΣΑΣ	Δε χρησιμοποιώ μπαστούνι ή πατερίτσες. (5) Χρησιμοποιώ μπαστούνι ή πατερίτσες και λίγο βάρος στο πονεμένο πόδι. (2) Δεν μπορώ να βάλω βάρος στο πονεμένο πόδι. (0)
ΕΝΟΤΗΤΑ 3: ΑΙΣΘΗΣΗ ΟΤΙ ΚΛΕΙΔΩΝΕΙ ΤΟ ΓΟΝΑΤΟ	Δεν αισθάνομαι καθόλου ότι κλειδώνει ή ότι πιάνεται το γόνατο μου. (15) Αισθάνομαι ότι το γόνατό μου πιάνεται αλλά δεν κλειδώνει. (10) Το γόνατο μου κλειδώνει περιστασιακά. (6) Το γόνατο μου κλειδώνει συχνά. (2) Νοιώθω το γόνατο μου κλειδωμένο αυτή τη στιγμή. (0)
ΕΝΟΤΗΤΑ 4: ΑΙΣΘΗΣΗ ΟΤΙ ΦΕΥΓΕΙ ΤΟ ΓΟΝΑΤΟ	Το γόνατο μου δεν φεύγει ποτέ. (25) Το γόνατο μου φεύγει σπανίως, μόνο κατά τη διάρκεια αθλητικών ή επίπονων δραστηριοτήτων. (20) Το γόνατο μου φεύγει συχνά σε αθλητικές ή επίπονες δραστηριότητες. Κατά συνέπεια δεν μπορώ να συμμετέχω σε τέτοιες δραστηριότητες. (15) Το γόνατο μου φεύγει συχνά κατά τη διάρκεια καθημερινών δραστηριοτήτων. (10) Το γόνατο μου φεύγει πολύ συχνά κατά τη διάρκεια καθημερινών δραστηριοτήτων. (5) Το γόνατο μου φεύγει σε κάθε βήμα που κάνω. (0)
ΕΝΟΤΗΤΑ 5: ΠΟΝΟΣ	Δεν έχω πόνο στο γόνατο μου. (25) Έχω διαλείποντα ή ήπιο πόνο στο γόνατο μου κατά τη διάρκεια επίπονων δραστηριοτήτων. (20) Έχω έντονο πόνο στο γόνατό μου κατά τη διάρκεια επίπονων δραστηριοτήτων. (15) Έχω έντονο πόνο στο γόνατό μου κατά τη διάρκεια ή αφού περπατήσω περισσότερο από ένα μίλι (περίπου 1.5 χιλιόμετρο). (10) Έχω έντονο πόνο στο γόνατό μου κατά τη διάρκεια ή αφού περπατήσω λιγότερο από ένα μίλι (περίπου 1.5 χιλιόμετρο). (5) Έχω συνεχή πόνο στο γόνατό μου. (0)
ΕΝΟΤΗΤΑ 6: ΠΡΗΞΙΜΟ (ΟΙΔΗΜΑ)	Δεν έχω πρήξιμο (οίδημα) στο γόνατό μου. (10) Έχω πρήξιμο (οίδημα) στο γόνατό μου μόνο μετά από επίπονες δραστηριότητες. (6) Έχω πρήξιμο (οίδημα) στο γόνατό μου μετά από συνηθισμένες δραστηριότητες. (2) Το γόνατό μου είναι συνέχεια πρησμένο. (0)
ΕΝΟΤΗΤΑ 7: ΑΝΑΒΑΣΗ ΣΚΑΛΑΣ	Δεν έχω προβλήματα ανεβαίνοντας σκάλες. (10) Έχω ήπια προβλήματα όταν ανεβαίνω σκάλες. (6) Μπορώ να ανέβω μόνο ένα-ένα τα σκαλοπάτια. (2) Δεν μπορώ να ανεβώ σκάλες. (0)
ΕΝΟΤΗΤΑ 8: ΒΑΘΥ ΚΑΘΙΣΜΑ	Δεν έχω προβλήματα στο βαθύ κάθισμα. (5) Έχω ήπια προβλήματα στο βαθύ κάθισμα. (4) Δεν μπορώ να κάνω βαθύ κάθισμα πάνω από 90 μοίρες. (1) Δεν μπορώ να κάνω καθόλου βαθύ κάθισμα λόγω του γόνατος μου. (0)
ΣΥΝΟΛΟ	…………… / 100

Appendix B: Greek version of the Tegner Activity Scale

**Table 6:** Greek Tegner Activity Scale.

ΕΡΩΤΗΜΑΤΟΛΟΓΙΟ TEGNER Παρακαλώ σημειώστε παρακάτω το ΜΕΓΑΛΥΤΕΡΟ επίπεδο δραστηριότητας στο οποίο μπορείτε να συμμετέχετε ΑΥΤΗ ΤΗ ΣΤΙΓΜΗ:	
ΕΠΙΠΕΔΟ 10	Ανταγωνιστικά αθλήματα: Ποδόσφαιρο (εθνική και διεθνής ελίτ)
ΕΠΙΠΕΔΟ 9	Ανταγωνιστικά αθλήματα: Ποδόσφαιρο (πιο μικρές κατηγορίες), Πάλη, Ενόργανη γυμναστική
ΕΠΙΠΕΔΟ 8	Ανταγωνιστικά αθλήματα: Μπάντμιντον, Στίβος (άλμα εις ύψος κτλ.), Σκι (κατάβασης)
ΕΠΙΠΕΔΟ 7	Ανταγωνιστικά αθλήματα: Τένις, Στίβος (τρέξιμο), Μοτοκρος (αγώνες ταχύτητας), Χάντμπολ, Μπάσκετ Ερασιτεχνικά αθλήματα: Ποδόσφαιρο, Μπάντμιντον, Στίβος (άλμα εις ύψος κτλ.), Αγώνες στίβου ανώμαλου δρόμου ως άθλημα αναψυχής και σε επαγγελματικό επίπεδο
ΕΠΙΠΕΔΟ 6	Ερασιτεχνικά αθλήματα: Τένις και μπάντμιντον, Χάντμπολ, Μπάσκετ, Σκι (κατάβασης), Τζόκινγκ (ελαφρύ τρέξιμο) τουλάχιστον 5 φορές την εβδομάδα
ΕΠΙΠΕΔΟ 5	Εργασία: Βαριά εργασία (π.χ. οικοδόμος, αγρότης) Ανταγωνιστικά αθλήματα: Ποδήλατο, Σκι σε μη οργανωμένη πίστα Ερασιτεχνικά αθλήματα: Τζόκινγκ (ελαφρύ τρέξιμο) σε ανώμαλο έδαφος τουλάχιστον 2 φορές την εβδομάδα
ΕΠΙΠΕΔΟ 4	Εργασία: Μετρίως βαριά εργασία (π.χ. οδηγός νταλίκας, βαριά οικιακή εργασία) Ερασιτεχνικά αθλήματα: Ποδήλατο, Σκι σε μη οργανωμένη πίστα, Τζόκινγκ (ελαφρύ τρέξιμο) σε ομαλό έδαφος τουλάχιστον 2 φορές την εβδομάδα
ΕΠΙΠΕΔΟ 3	Εργασία: Ελαφριά εργασία Ανταγωνιστικά και ερασιτεχνικά αθλήματα: Κολύμβηση Περπάτημα στο δάσος/εξοχή δυνατό
ΕΠΙΠΕΔΟ 2	Εργασία: Ελαφριά εργασία Περπάτημα σε ανώμαλο έδαφος δυνατό αλλά περπάτημα στο δάσος/εξοχή αδύνατο
ΕΠΙΠΕΔΟ 1	Εργασία: Καθιστική εργασία Περπάτημα σε ομαλό έδαφος δυνατό
ΕΠΙΠΕΔΟ 0	Αναρρωτική άδεια ή σύνταξη αναπηρίας λόγω των προβλημάτων στο γόνατο
